# A realist review of shared medical appointments: How, for whom, and under what circumstances do they work?

**DOI:** 10.1186/s12913-017-2064-z

**Published:** 2017-02-04

**Authors:** Susan R. Kirsh, David C. Aron, Kimberly D. Johnson, Laura E. Santurri, Lauren D. Stevenson, Katherine R. Jones, Justin Jagosh

**Affiliations:** 1 Louis Stokes Cleveland VA Medical Center, Cleveland, OH USA; 2 School of Medicine, Case Western Reserve University, Cleveland, OH USA; 3 Weatherhead School of Management, Case Western Reserve University, Cleveland, OH USA; 4 College of Nursing, University of Cincinnati, Cincinnati, OH USA; 5 Frances Payne Bolton School of Nursing, Case Western Reserve University, Cleveland, OH USA; 6 Centre for the Advancement of Realist Evaluation and Synthesis, University of Liverpool, Liverpool, UK

**Keywords:** Shared medical appointments, Group medical visits, Realist review

## Abstract

**Background:**

Shared medical appointments (SMAs) are doctor-patient visits in which groups of patients are seen by one or more health care providers in a concurrent session. There is a growing interest in understanding the potential benefits of SMAs in various contexts to improve clinical outcomes and reduce healthcare costs. This study builds upon the existing evidence base that suggests SMAs are indeed effective. In this study, we explored how they are effective in terms of the underlying mechanisms of action and under what circumstances.

**Methods:**

Realist review methodology was used to synthesize the literature on SMAs, which included a broad search of 800+ published articles. 71 high quality primary research articles were retained to build a conceptual model of SMAs and 20 of those were selected for an in depth analysis using realist methodology (i.e.,middle-range theories and and context-mechanism-outcome configurations).

**Results:**

Nine main mechanisms that serve to explain how SMAs work were theorized from the data immersion process and configured in a series of context-mechanism-outcome configurations (CMOs). These are: (1) Group exposure in SMAs combats isolation, which in turn helps to remove doubts about one’s ability to manage illness; (2) Patients learn about disease self-management vicariously by witnessing others’ illness experiences; (3) Patients feel inspired by seeing others who are coping well; (4) Group dynamics lead patients and providers to developing more equitable relationships; (5) Providers feel increased appreciation and rapport toward colleagues leading to increased efficiency; (6) Providers learn from the patients how better to meet their patients’ needs; (7) Adequate time allotment of the SMA leads patients to feel supported; (8) Patients receive professional expertise from the provider in combination with first-hand information from peers, resulting in more robust health knowledge; and (9) Patients have the opportunity to see how the physicians interact with fellow patients, which allows them to get to know the physician and better determine their level of trust.

**Conclusions:**

Nine overarching mechanisms were configured in CMO configurations and discussed as a set of complementary middle-range programme theories to explain how SMAs work. It is anticipated that this innovative work in theorizing SMAs using realist review methodology will provide policy makers and SMA program planners adequate conceptual grounding to design contextually sensitive SMA programs in a wide variety of settings and advance an SMA research agenda for varied contexts.

## Background

A shared medical appointment (SMA) is a clinical encounter in which a group of patients receive patient education and counseling, physical examination, and clinical support in a group setting [1, 2]. Typically SMAs are designed to have one or more health care provider(s) attend to a group of patients who share a common illness or demographic make-up. In contrast to group education alone, patients in SMAs engage in care that may include a physical exam, medication adjustments or other clinical interventions that are tailored to the needs of the group as well as the individual patients. In contrast to one-on-one visits, SMAs provide a longer appointment timeframe as well as the opportunity for patients to witness patient-provider interactions and share in peer support. SMAs are consistent with the chronic care model of Wagner et al., a model which informed our own conceptualization and implementation of SMAs [3, 4].

Interest in SMA interventions has been increasing due to the potential for enhancing the quality of healthcare, mitigating health disparities, improving self-management, and cost-saving [5, 6]. Previous systematic reviews of SMAs have mainly focused on the question of efficacy [6, 7]. Although both primary studies and systematic reviews have demonstrated efficacy “on the average”, results from individual studies are quite varied [6–9]. In addition, a search through the literature has revealed many studies involving SMAs (now >100) that demonstrate significant diversity in terms of implementation context, intervention approach, and assessment methodology [3, 10–67]. Although a variety of explanations have been offered about how SMAs work, there has been relatively little robust middle-range programme theory development that would allow better targeting of SMA interventions to patients and contexts where it is most likely to be effective. Our objective in using the realist approach was to identify underlying causal mechanisms and explore how they work under what conditions, i.e., developing and refining theory of SMAs, accounting for context as well as outcomes [68]. In so doing, we sought to provide evidence-informed programme theories to inform the implementation of SMAs in a variety of settings.

## Methods

### Realist review (a glossary of terms is included in Table 1)

**Table 1:** Glossary of Terms

Realism: The philosophy of realism brings attention to the limits of both logical empiricism which obfuscates the active theorizing of unobservable agents of causation (e.g., as demonstrated through the logic of randomized controlled trials) and constructivism which negates the belief of universal laws in favor of comparing storylines and paradigms. Realist modes of research reflect a mix of these two approaches by posing the kinds of questions that seek out the truth of matters, while at the same time operating from a view of the context bound and contingent nature of human knowledge.
Realist Review (RR): is a theory-driven approach to synthesizing quantitative, qualitative or mixed methods research, from a perspective based in Realism. It answers questions of the general format ‘what worked, for whom and in what circumstances, how and why?’ The basis of a realist causal explanation is Context + Mechanism = Outcome (Otherwise referred to as the CMO configuration)
Middle-range theory (MRT): Middle-range theory is an implicit or explicit theory that can used to explain the cause of outcomes for programs and interventions or parts thereof. “Middle- range” means that the theory can be tested with the observable data and is not abstract to the point of addressing larger social or cultural forces (i.e., grand theories) [1]. MRT is formulated at the outset of a realist review and examined in relation to empirical evidence throughout the review process.
Context-mechanism-outcome (CMO) configurations: CMO configuring is a heuristic used to generate causative explanations pertaining to outcomes in the observed data. The process draws out and reflects on the relationship of context, mechanism, and outcome of interest in a particular program. A CMO configuration may pertain either to the whole program or only to certain aspects.
Context: Context often pertains to the “backdrop” of programs and research. As conditions change over time, the context may reflect aspects of those changes while the program is implemented. Examples of context include cultural norms and history of the community in which a program is implemented, the nature and scope of existing social networks, or built program infrastructure. They can also be trust-building processes, geographic location (e.g., rural or urban), types of funding sources, and other opportunities or constraints.
Mechanism: A mechanism is the generative force that leads to outcomes. It typically denotes the reasoning (cognitive or emotional) of the various actors in relation to the work, challenges, and successes of the partnership. Mechanisms are linked to, but not synonymous with, the program’s strategies (e.g., a strategy may be an intended plan of action, whereas a mechanism involves the participants’ reaction or response to the intentional offer of incentives or resources). Identifying the mechanisms advances the synthesis beyond describing “what happened” to theorizing “why it happened, for whom, and under what circumstances.”
Outcomes: Outcomes are either intended or unintended and can be proximal, intermediate, or final. Examples of intervention outcomes are improved health status, increased use or quality of health services, or enhanced research results.
Demi-regularity: Demi-regularity means semi-predictable patterns or pathways of program functioning. The term was coined by Lawson, who argued that human choice or agency manifests in a semi-predictable manner—“semi” because variations in patterns of behavior can be attributed partly to contextual differences from one setting to another [2].

Realist Review methodology was chosen to uncover *how* and *for whom* and *under what circumstances* SMAs work. The general pattern of work in realist review involves a set of iterative (i.e., non-linear) steps involving: (a) establishing research questions; (b) identifying candidate middle-range theory(ies) (MRT) for the program; (c) searching the literature to select, appraise, and retain primary studies and scan for new candidate MRTs; (d) synthesizing evidence using context-mechanism-outcome configurations (CMOc); and (e) refining the evidence-based middle-ranged theories to answer questions related to ‘what works, for whom, under what circumstances and how.’[69, 70] Middle-range theory should embody the underlying logic of the program in question (across many cases), and may also include causal theories on the key elements of context, mechanism and outcome. The synthesis stage involves examining the MRT in relation to the evidence captured, through a heuristic process of constructing, exploring, and refining context-mechanism-outcome configurations. Data to include in CMO configurations can involve primary outcomes (quantitative or qualitative) as well as program and setting descriptions and interpretation of outcomes by study authors [71].

Context can include social as well as physical settings, cultural/psychological norms, demographic factors, institutional practices and so on. Our definition of mechanism relies on the description laid out by Pawson in 2006, to include the cognitive and/or emotional responses of participants to resources offered [71]. For this review, we define ‘mechanisms’ as the way in which patients react/respond to the group clinical encounter, with specific attention paid to the range of resources offered through SMAs. Thus the CMO configurations portray how SMA programs works and under what contextual conditions. Further detail about realist synthesis methodology has been published [68, 72, 73]. We note that in contrast to Cochrane reviews which involve identification of the review question(s) and search for primary studies using pre-defined inclusion and exclusion criteria, realist review is an iterative process that involves clarification and refinement of the scope of the review and search for relevant evidence, refining inclusion criteria in the light of emerging data [69, 70]. In contrast, to the focus of Cochrance reviews on relevance to the specific research question and methodological rigor, a realist approach considers relevance and rigor from a broader fitness for purpose perspective.

### Overview of the research team

The review team represented a variety of disciplines and professions (medicine, nursing, and social sciences) as well as experience in implementation research (DCA, SK, LDS), management research (DCA, JO), program evaluation (KJ, LES, LDS), systematic reviews (KJ,KRJ), chronic care delivery (DCA, SK, KJ, LES, LDS), and realist synthesis (JJ). SK is the national VA subject matter expert and along with DCA has been conducting and studying SMAs since 2006. SK and DCA formed the initial review questions and led the search, selection and appraisal of papers. LDS, LES, KDJ, and KRJ contributed initial study design and provided feedback on iteratively developed protocols. JJ joined at a later stage to further develop the middle-range theory and lead an in depth examination of 20 SMA studies, using CMO configurations.

### Literature search strategy and article retention

**Fig. 1:**
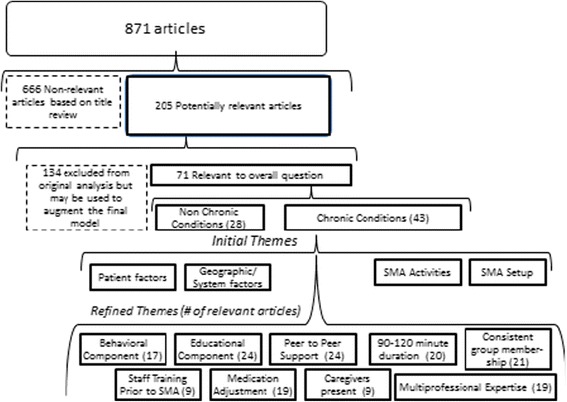
Literature Search Scheme

**Fig. 2:**
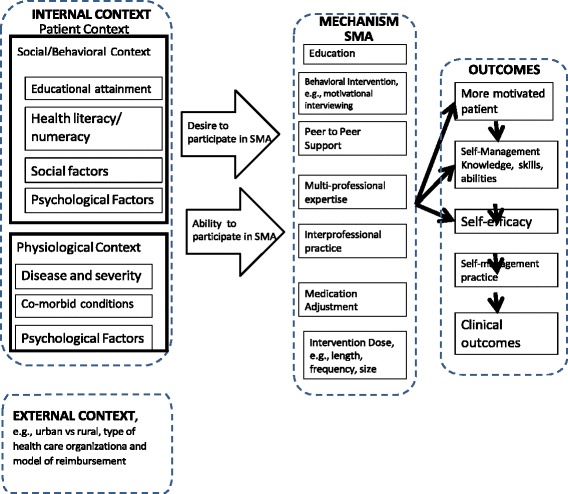
Final Conceptual Model. Dotted lines surround the three major components of the CMO Configuration: Context (Internal and External), Mechanism, and Outcome

The literature search was assisted by a librarian at the Health Sciences Library at CWRU. An initial search completed in September 2011 included Medline, CINAHL, and Pub Med using the following search terms: 1) shared medical appointments, 2) shared medical visits, 3) group appointments 4) group medical visits, 5) cluster visits, 6) chronic care clinics, 7) primary care group visit, 8) group medical appointment, and 9) group office visits. The search time frame was designed to coincide with a parallel evidence based synthesis using a traditional approach to systematic review that involved meta-analysis and statistical regression; both projects supported by funding provided by VA HSR&D, but were conducted independently [7]. The results of a search of Pub Med, Medline, and CINAHL are diagramed in Fig. 1. Eight hundred seventy one citations were reviewed with 205 meeting criteria (based on inclusion of terms relevant to SMAs in the article title). Of 205 articles, each was assessed by two independent abstractors using an adapted data collection tool based on the instrument of Zaza et al. [74] This standardized abstraction form and procedure was chosen to enhance consistency, reduce bias, and improve validity and reliability. Discrepancies between reviewers were resolved by consensus (The abstraction form and grid are available from the authors). Through this rigorous selection and appraisal process, 71 high quality, relevant SMAs articles were retained and examined in relation to the conceptual models of SMAs that were developed prior to the review [3] and which evolved during its initial stages; our final model is shown in Fig. 2. At this point, co-author (JJ) was invited to join the team as a realist review expert and to continue with the analysis of 71 articles. We then used 20 articles, purposively sampled to achieve a representative sample of types of sites, patients, and geography to conduct an in depth analysis involving CMO configurations. (See Table 2) The CMO configuration analysis followed the construction of candidate middle-range program theories – the latter being constructed from the expertise of the co-investigative team and a general reading of 71 articles. In constructing the CMO configurations, a series of middle-range program theories were hypothesized to suggest how SMAs work to improve service delivery and self-efficacy. The purposively selected 20 articles were read and re-read with an eye to coding data that provided support to these theories. After coding, exemplar quotes (i.e., that had the most explanatory power) were retained for synthesis using CMO configuration. To ensure transparency and rigor of the findings, these exemplar quotes were extracted and a CMO configuration was formulated in relation to each quotation. In most cases when authors reported an explanatory claim about how SMAs function to produce outcomes, an overt connection between context, mechanism and outcome was not made. Thus the CMO configurations represent our work in theorizing the connection between context, mechanism, and outcome, based on the quotes. The CMO configurations were thus inferred both from the literature and subject area expertise of the co-investigators, to become a set of evidence-informed theoretical claims for explaining how SMAs work.

**Table 2:** Articles Retained for Realist Synthesis

A.	Due-Christensen et al. [29]. Can sharing experiences in groups reduce the burden of living with diabetes, regardless of glycemic control?
B	Culhane-Pera et al. [23]. Group visits for Hmong adults with type 2 diabetes mellitus.
C	Clancy et al. [18] Further Evaluating the Acceptability of Group Visits in an Uninsured Population with Diabetes .
D	Sadur et al. [50] Diabetes Management in a Health Maintenance Organization.
E	Trento et al. [61]. A 5-Year randomized controlled study of learning, problem solving ability, and quality of life modification in people with type 2 diabetes managed by group care.
F	Taveira et al. [57]. Pharmacist-led group medical appointments for the management of type 2 diabetes with comorbid depression in older adults.
G	Kirsh et al. [3]. Shared medical appointments based on the chronic care model: a quality improvement project to address the challenges of patients with diabetes with high cardiovascular risk.
H	De Vries et. al. [25]. Implementation and outcomes of group medical appointments in an outpatient specialty care clinic.
I	Harris, M. [35]. Shared Medical Appointments after Cardiac Surgery - The process of Implementing a Novel Pilot Paradigm to Enhance Comprehensive Post Discharge Care.
J	Miller et al. [45]. Group Medical Visits for Low-Income Women with Chronic Disease.
K	Meehan et al. [44]. GMA -Organization and Implementation in the Bone Marrow Transplantation Clinic.
L	Kawasaki L et al. [39] Willingness to attend group visits for hypertension treatment.
M	Shojania K, Ratzlaff M. [54] Group visits for rheumatoid arthritis patients: a pilot study.
N	Bray P et al. [14]. Confronting disparities in diabetes care: the clinical effectiveness of redesigning care management for minority patients in rural primary care practices .
O	Geller JS et al. [67] Impact of a group medical visit program on Latino health-related quality of life.
P	Naik AD et al. [46]. Comparative effectiveness of goal setting in diabetes mellitus group clinics. Randomized controlled trial.
Q	Lavoie JG et al. [40]. Group medical visits can deliver on patient centred care objectives: results from a qualitative study.
R	Cohen S et al. [20] Veteran experiences related to participation in shared medical appointments.
S	Esden JL, Nichols MR. [33] Patient-centered group diabetes care: a practice innovation.
T	Vachon GC et al. [64] Improving access to diabetes care in an inner-city, community-based outpatient health center with a monthly open-access, multistation group visit program.

#### Middle range theory

The middle-range program theories for this review were guided by the question ‘What is the intrinsic logic of SMA programs? (i.e., what explains why SMAs are assumed to be a good idea?)’ Our MRT work was built from key insights published in primary study literature reporting SMA findings and co-investigator expertise. Specifically, these theories were consistent with evidence from studies conducted in settings other than SMAs on chronic disease education, self-management, stigma, and peer support. [75–83] For example, in our sample, Due-Christensen et al. [29] have noted that “psychological burden and difficulty in accepting a diagnosis of diabetes can lead to patients attempting to normalize the situation by concealing their condition, which makes self-management difficult (p. 251).” Similarly a patient quote in Lavoie et al. [40] states: “With a group you have a feeling of being part of many, whereas when I’m here with you or with my doctor, or one-on-one, quite often you’re intimidated by someone who knows more than you do and it’s just a feeling sometimes of isolation and loneliness because you have the disease and it’s a different feeling completely. And I feel a lot more comfortable in groups than one-on-one [40]. (p. 4)” The co-investigative team believed these statements to reflect the intrinsic logic of SMA programs and a number of linked MRT statements were formulated across a number of premises hypothesizing how SMAs work and for whom:

- Diagnosis and hardship from illness burdens a person, both physically and mentally, activating a natural urge to normalize or conceal the illness.
- Normalizing or concealing an illness hinders pro-active self-management because of the difficulty in taking care of health issues that are unacknowledged or remain hidden.
- One-on-one visits with clinical providers (ideally) provide diagnoses, education, intervention, and support. However, the relatively private nature of the one-on-one visit does not safeguard against the natural reaction of many patients to normalize or concealing the illness situation.
- SMAs provide resources similar to one-on-one consultations, with the added element of communal disclosure of one’s illness, altered doctor-patient dynamics, and peer-to-peer support. Thus, patients experience new resources and opportunities in the following ways:
- Patients experience their illness through the eyes of peers. Such communal disclosure of illness leads to a reduction of normalization and concealment, which in turn leads to improved self-management.
- Patients meet other patients with similar health issues, which offer something to which they can compare their situation. Such comparisons can lead to increased motivation for self- management through being inspired by others, or else decreased motivation by feeling inadequate in comparison to others.
- Patients offer support and advice to other patients, which can help them feel positive about their position as peer role models, leading them to feel a healthy sense of pressure to maintain standards for their own care.
- Patients have the opportunity to listen to the health care provider speak to other patients with similar conditions, thus increasing the opportunity to hear and absorb important information about illness management.
- Not all patients normalize or conceal their medical conditions. Thus it is suspected that the resource that SMAs offer in terms of communal disclosure of illness will show positive outcomes for patients who tend to normalize and conceal their condition, and maybe less impactful on independent or self-motivated patients who may find ‘coming out’ to a group to increase, rather than decrease stress.
- There may be class, race and gender issues with certain cultural groups that interfere with the potential benefit of public disclosure of illness in clinical setting. SMAs for particular cultural groups may not work if tight-knit communities require greater anonymity in the clinical encounter to prevent community-based stigma. This may further vary according to SMA type. An SMA for diabetes may have less stigma, for example, than one for mental health. This set of middle-range program theory propositions have guided our realist analysis involving CMO configurations (CMOcs), and are further discussed in the discussion section.

### Results

The findings are organized across 9 sections describing the key mechanisms and contexts to explain how SMAs work and for whom. These are presented below and depicted in summary form in Table 3. These CMOcs were constructed by the research team, based on an interpretation of the key explanatory quotations in the context of evidence from other papers within the twenty reviewed. Within each section, one or more quotations from the primary literature are presented, followed by a context-mechanism-outcome configuration that provides an explanatory interpretation of the quotation. References from the other most relevant papers are cited. The sections are titled as followed:

1. Group exposure combats isolation, which in turn helps to remove doubts about one’s ability to manage illness.
2. Patients learn about disease self-management vicariously by witnessing others’ illness experiences.
3. Patients feel inspired by seeing others who are coping well.
4. Group dynamics lead patients and providers to develop more equitable relationships.
5. Providers feel increased appreciation and rapport toward colleagues leading to increased efficiency.
6. Providers learn from the patients how better to meet their patients’ needs.
7. Adequate time allotment of the SMA leads patients to feel supported.
8. Patients receive professional expertise from the provider in combination with first-hand information from peers, resulting in more robust health knowledge.
9. Patients have the opportunity to see how the physicians interact with other patients, which allows them to get to know the physician and better determine their level of trust.

**Table 3:** Summary of CMO configurations

CMOc Subsection	Context + Mechanism = Outcome		
1. Combats Isolation	Isolation	Social contact (resource) → Correcting misperceptions(response)	Likely improved in self-efficacy
2. Vicarious Learning	Isolation	Exposure to others’ illness (resource) → gaining perspective on one’s illness situation (response)	Likely improved self-efficacy
3. Feeling inspired by successful peers	Low/high motivation for self-management behavior	Exposure to others’ successes (resource) → trying to emulate success (response)	Likely improved self-efficacy
4. Friendships develop between patients and providers	SMAs are more relaxed than one-on-one clinical encounters	New patient-provider friendships developed (resource) → fostering trust amongst all parties (response)	Likely improved motivations and self-efficacy
5. Improved collegiality amongst providers	Providers typically work in isolation	Team members are able to witness and interact (resource) → leading to mutual appreciation of respective roles and bonding (response)	Likely improved service delivery and work satisfaction
6. Provider learning	Providers unaware of patient needs	Group setting encouraged creative thinking about meeting people’s needs	Likely improved service delivery
7. Adequate time allotment	SMAs are longer sessions than one-on-one clinical visits	Allows patients and providers to get to know each other, relax (resource) → leads to a sense of comfort for the patient (response)	Likely improved self-management
8. First-hand health knowledge	Isolation	Group visit allows patients to share, confirm/dispute information (resource) → leads to patients feeling reassured about health knowledge provided (response)	Likely improved application of information given
9. Increased trust in physician	Mistrust of physicians a common experience in healthcare	SMA creates more even power dynamics between patient and provider (resource) → leads to patient feeling increased trust in physician (response)	Improved doctor-patient relationship and likely improved self-efficacy

[85] Merton RK. Social Theory and Social Structure. New York: Simon and Schuster, 1968

[86] Lawson T. Economics and Reality. London: Routledge, 1997

### CMOc 1: group exposure combats isolation, which in turn helps to remove doubts about one’s ability to manage illness [21, 25, 29, 33, 35, 40, 54]

Typically, patients are isolated in their illness experience. This often leads to a number of problems including cognitive dissonance and misperception of one’s situation and abilities. The SMA created social contact amongst people with similar background or illness, which relieved these misperceptions:

‘you have a feeling of not being capable enough, you feel you are the only one who is not able to manage it, everyone else is capable for sure, but I do not know anyone else. Therefore you get relaxed when you meet others who feel the same way.’ [29] (p. 253)

CMO configuration (CMOc): Feelings of inadequacy prevailed coupled sometimes with isolation (context). The SMA created social contact amongst a group of people with similar illness experiences. This exposure helped to correct misperceptions about their capabilities and the capabilities of others in self-efficacy (mechanism). The social contact combined with people sharing similar experience contributed to esprit de corps which promoted self-efficacy (outcome).

### CMOc 2: patients in SMAs learn about disease self-management vicariously by witnessing others’ illness experiences [21, 25, 35, 44, 54]

Patients found it eye-opening to witness the illness experience of others. This had an impact on their comfort-level, knowing that they are not alone, and learning about the illness through the experience of others:

‘Many patients had no inkling that others were experiencing the same or similar symptoms and stated that they were often comforted knowing that they were not alone in dealing with their post-cardiac surgery problems…Having others present in the conference room who had similar surgeries or surgeons sparked a lot of lively discourse among the participants, as they were curious to know if they were ambulating as well or had similar amounts of pain, wound issues, or musculoskeletal symptoms after discharge.’ [35] (p. 128)

‘all patients highlighted the knowledge they had gained from other patients who shared similar issues and concerns.’ [44] (p. 88)

CMOc: Patients typically experience their illness in isolation (context). The group visit offered patients the opportunity to compare their illness experience with the experience of others who are in similar situations. Patients were curious to know how other patients were managing, because such comparisons help to understand one’s own illness experience (mechanism). This most likely led to improved self-efficacy and reduction of stress, supporting disease management and healing (outcome).

### CMOc 3: patients feel inspired by seeing others who are coping well [21, 25, 35]

The mix of patients in SMAs brought out key group dynamics which supported self-efficacy. Some patients were doing better than others, which triggered two separate mechanisms. For those who were doing better, their role modeling served to keep them motivated to stay on track. For those who were doing less well, witnessing others who were progressing well served as motivation and something to strive for:

‘…Some participants set high goals for physical activity and this encouraged other members. They also had the opportunity to share their knowledge and had a feeling of usefulness or altruism’ [25]. (p. 5)

“There used to be a guy here…he lost like 40 some pounds and we really clapped for that fellow cause he really worked hard for that. And it gave us something to try for.” [21] (p. 1289)

**CMOc:** SMA brought patients together, some of whom had lower motivation for self- management behavior and others who had relatively higher motivation (context). This led to feelings of usefulness and altruism (mechanism) for the former group, and motivation to do better for the latter (mechanism). Either way, these mechanisms most likely led to improved self-efficacy and disease management (outcome).

### CMOc 4: group dynamics lead patients and providers to develop more equitable relationships [21, 33, 45, 54]

There was some evidence that SMAs alter patient-provider relationships such that friendship and partnerships were formed between patients and providers. Although there was not much detail on the inner mechanisms of this effect, we speculate that having multiple patients in a room, and increased duration of the clinical encounter helped to balance the power dynamic between providers and patients and allowed the patient to get to know the provider. One patient noted that the SMA helped in better understanding the doctor. This was perhaps due to the fact of witnessing the doctor in action with other patients:

‘A number of the study participants expressed the concept of becoming friends with the GMV provider. The informal structure of the GMV may have allowed the patient and provider to develop a partnership rather than having a more traditional active-passive relationship.’ [45] (p. 223).

‘I got to meet new people and really get a feel for my doctor.’ [33] (p. 47)

**CMOc:** Because of the structure of group visits with many patients involved, the clinical encounters are more informal, relaxed and friendly than one-on-one clinical encounters (context). This allowed new patient-provider relationships, including friendships, which fostered trust amongst all parties (mechanism). Stronger, more trusting relationships between patients and provider was a result (outcome)

### CMOc 5: providers feel increased appreciation and rapport toward colleagues leading to increased efficiency [3, 40, 44, 50]

In some SMAs, a number of providers worked together to provide clinical care. There was some evidence that the experience of providers witnessing other providers with patients, and working together in SMAs increased team cohesion and coordination of service. This led to increased work satisfaction and enhanced working relationships:

‘We have discovered a high level of satisfaction and enhanced team-building among the transplantation care providers. This type of appointment is a team-based approach to care, with each member of our bone marrow transplantation team playing an important role in the overall success of the program. This not only led to increased efficiency and team rapport but also to a mutual appreciation for the helpful role that each team member plays in the process …Because this type of visit is so novel, there is increased physician satisfaction. The team approach to health care also offers care providers with the help, encouragement, and assistance of an entire care delivery team, which rapidly evolves into a cohesive, goal oriented health-care team’ [44] (p. 89–90)

**CMOc:** Typically providers work in isolation with patients (context). The SMA, in which team members could interact with each other and gain mutual appreciation of their roles increased bonding, and mutual appreciation (mechanism). This led to improved service delivery and work satisfaction. Staff felt supported and collectively created a more cohesive team (outcome)

### CMOc 6: providers learn from the patients how better to meet their patients’ needs [3, 40, 50]

SMAs were described by some service providers as an environment for increased learning on how to improve service delivery, due to the fact that patients in the room were sharing and brainstorming ideas about illness management, from which the service provider gleaned new ideas. In the following quotation, the service provider suggested that this would not have been possible in a one-on-one visit. This may be due to a variety of reasons, including the fact that the one-on-one visit doesn’t as easily open up such kinds of brainstorming and information sharing:

“I think that it [the GMV {sic – equivalent to SMA}] has helped me to be more creative in looking at ways to meet people’s needs. Some of that just comes from the patients themselves because they often have some really neat ideas about how to overcome challenges or difficulties in dealing with the diabetes. So I think that, not only have I become more aware but I’ve also, they’ve given me some really good tips and ideas. I think there’s stuff I learned that I wouldn’t have learned if I had done it on an individual basis. There’s a lot of value that comes out of that, that kind of impromptu patient teaching of each other” Provider #28 [40]. (p. 5–6)

**CMOc:** The SMA created an environment in which patients with a common illness experience shared information and brainstormed ideas about self-management (context). This environment allowed the service provider to appreciate new ways of thinking about how to serve the patient population (mechanism). This in turn most likely led to improved service provision (outcome)

### CMOc 7: adequate time allotment of the SMA led patients to feel supported [40, 44, 46]

SMAs are typically longer than one-on-one appointments. The increased time that provider and patients spend together was reported as having a positive impact on patient perceptions:

‘We have discovered that patients are often encouraged and more hopeful when they spend time with other patients with similar or worse conditions’. Patients spend more time with their care providers and the specialty team in this setting. This provides a sense of comfort to each patient [44]. (p. 89)

**CMOc:** Due to having multiple patients in the clinical encounter, SMAs require substantially longer timeframes than regular visits (context). This added time allows patients and providers the opportunity to really get to know each other and have patients open up and relax in the clinical setting. This has been identified as comforting to the patient (mechanism). This relaxation and comfort presumably leads to better self-management (outcome).

### CMOc 8: patients received professional expertise from the provider in combination with first-hand information from peers, resulting in more robust health knowledge [21, 25, 44, 54]

Patients were able to absorb information from the provider in a more effective way due to the fact that such information was complemented by first-hand experience of other patients. This relieved stress and resolved questions that the provider was not always able to address:

During one of the GMAs (sic. Group Medical Appointments are equivalent to SMAs), a patient asked about travelling with oxygen and another patient explained how easy it was to travel. The NP may have been able to produce the same information but not the firsthand experience and the reassurance that it would not be complicated.’ [25] (p. 5)

**CMOc:** The SMA allows health information to be shared not only by the health professional, but also by other patients through firsthand experience (context). Patients receiving such knowledge felt reassured by the experience of other patients (mechanism). This likely led to decreased stressed, and improved application of the information given during sessions (outcome).

### CMOc 9: patients have the experience of witnessing the physician interact with fellow patients, which allows them to get to know the physician better and determine levels of trust. [3, 21, 40, 67]

Some patients found that witnessing the physician in a group setting interacting with other patients help them to feel increased trust toward the physician and the healthcare system in general. This may be due to the fact that the group patient setting encourages the physician to remain open, willing and transparent in the face of many eyes watching. Even with the best of intentions, the one-on-one visit does not typically support such physician attitudes and behaviors:

“I’ve learned to trust him. I trust him more than I used to and that’s important, that bond of trust has to be there. I trust him more when I see that he’s open to learning and figuring out new things that are only happening in group dynamics” Patient #8 [40]. (p. 6)

“Do you know…what [SMA] helps me to see is what the physician, his devotion of trying to solve a health problem and trying to correct it. That actually reestablishes my faith in the medical system because you can see that they’re really devoted to trying to figure out really what is ailing you” Patient #13 [40]. (p. 5–6)

**CMOc**: Mistrust of physicians is pervasive in the health care setting (context). The SMA created an environment in which patients and providers are on more equal grounding. This led patients to feel that the physician is trustworthy (mechanism) leading to improved doctor-patient relationships and presumably improved health outcomes (outcome).

## Discussion

This realist review was an analysis of SMA interventions to explain how they work to produce outcomes. The development of our middle-range program theories on the impact of public disclosure of illness, in combination with the CMOc analysis should be taken as new, evidence-informed theoretical insights about SMAs which can serve to guide future planning, research on, and evaluation of such programs. Our approach is premised on the notion that in order to understand how SMAs work in different contexts and for different people it is necessary to have an understanding of the range of mechanisms which explain how they work in a general sense, which was achieved here. SMAs have impact because the social dynamic created through the group clinical encounter has been demonstrated to have advantages over the traditional one-on-one visit [40]. Such benefit can be summarized as allowing patients to have a social support base, first-hand knowledge sharing, improved uptake of health knowledge by patients, new and creative problem-solving by providers, increased time during the visit leading to improved trust in the doctor-patient dynamic, healthcare staff getting to know each other and deepening their collegiality, and all-round improvement in the social dynamics. Although there was very little direct research in the primary literature examining the ripple effect of these improvements to the clinical encounter, we hypothesize that improved health outcomes in terms of stress reduction and increased self-efficacy are a result. These linkages should be studied in future research.

These findings are in line with, and even move beyond the middle-range theories presented at the outset, about the impact of public disclosure of illness and the potential missed opportunities in one-on-one clinical encounters in patients with chronic illnesses not meeting patient clinical outcomes measures. SMAs help patients break from their cognitive dissonance pertaining to their illness, and coming out of concealing or normalizing their conditions [29]. What our evidence base did not cover was premises 4e and 4f of our middle-range theory, which attempts to theorize the ‘for whom’ question for SMAs. We suspect that SMAs do not work for everyone or for every cultural group and although we’ve speculated as to how this might manifest, there was little evidence in the literature to test these hypotheses. However, with the synthesis of this review, new research can be designed to address these questions. For example, we know that SMAs create conditions for publicly disclosing one’s illness experience [29]. Any instance in which such public disclosure is bad for the patient may result in negative outcomes. For some patients who already have high levels of self-efficacy and who are private by nature, the SMA environment may prove to be stressful in ways that private clinical encounters are not. The long history of group therapy for those with mental illness notwithstanding, some studies of SMAs excluded patients with mental illness, especially serious mental illness [30]. Similarly, for certain minority groups which exist in tight-knit communities the public disclosure of illness may prove to be stressful due to the fact that some people would not benefit from others in the community knowing about their illness situations. These ‘for whom’ questions are difficult to answer in a review such as this, because most if not all published pieces on SMAs show a positive publication bias, and do not actively research patients who choose not to involve themselves in SMAs.

Another limitation of this review is that it was not able to use successive data sources (i.e., qualitative and quantitative data) in CMO configurations to increase the robustness of findings. This is because, as a first attempt at an explanatory realist review of SMAs, the Middle-range theory work necessitated the extraction of literature evidence that provided explanatory claims. This was found in the qualitative findings and author interpretation of findings. It is another layer of analysis to link these explanatory CMOcs with the quantitative findings of SMA trials for example. As a direction for future research, it would be prudent to test these theories in light of both qualitative and quantitative outcomes. Our realist review complements findings from other approaches to “systematic” review. Realist and more traditional methods approach systematic review from different perspectives. Traditional, systematic review methods focus on program efficacy and effect size on the average. However, relevant evidence is often limited or conflicting [84]. They provide little or no assistance as to why the intervention worked or did not work when applied in different contexts or circumstances [71]. A realist review is context-dependent and theory-driven and provides an explanatory analysis aimed at discerning what works for whom, in what circumstances, in what respects, and how [71]. That is, the realist reviewer’s fundamental claim is that the effect of a mechanism (e.g., SMA’s mechanism of action) is contingent upon context (e.g., the type of site and type of patients). Thus, the realist researcher’s job focuses on identifying the contingencies between mechanism and contexts [68]. Theory development, which is an intrinsic part of realist review, can help identify new questions for further study and not just highlight gaps in the data for pre- existing conceptual models. In contrast to the theory-based approach of our realist review, Edelman et al. in their systematic review did not examine potential mechanisms. Rather, they used meta-regression to assess impact of specific structural aspects of SMAs, e.g., team continuity, length of SMAs, and whether or not breakout sessions were included [7]. Other reviews, when they conducted qualitative analyses, did not make explicit the theory linking data and conclusions nor were the pathways specified [6].

In summary, with this review, we have crafted new, original ideas about the functioning of SMAs, which we hope will serve this ever-growing area across many disciplines in clinical care.

## Conclusions

Our findings in this realist review suggest that the development of our middle-range program theories on the impact of public disclosure of illness, in combination with the CMOc analysis should be taken as new, evidence-informed theoretical insights about SMAs which can serve to guide future planning, research on, and evaluation of such programs. Our approach is premised on the notion that in order to understand how SMAs work in different contexts and for different people it is necessary to have an understanding of the range of mechanisms which explain how they work in a general sense, which was achieved here. advantages over the traditional one-on-one visit. Realist review offers an additional method to unpack the shared medical appointments and reveal underlying mechanisms and dynamics.
